# One-year costs of medical admissions with and without a 30-day readmission and enhanced risk adjustment

**DOI:** 10.1186/s12913-019-3983-7

**Published:** 2019-03-12

**Authors:** Sarah Zheng, Amresh Hanchate, Michael Shwartz

**Affiliations:** 1 University of Victoria Gustavson School of Business, 3800 Finnerty Rd, Victoria, BC V8P 5C2 Canada; 2 Boston University School of Medicine, 801 Massachusetts Ave Crosstown Center, Boston, MA 02118 USA; 3 Operations and Technology Management Department, Boston University Questrom School of Business, 595 Commonwealth Avenue, Boston, MA 02215 USA

**Keywords:** 30-day readmissions, Utilization, Disease severity

## Abstract

**Background:**

To overcome the limitations of administrative data in adequately adjusting for differences in patients’ risk of readmissions, recent studies have added supplemental data from patient surveys and other sources (e.g., electronic health records). However, judging the adequacy of enhanced risk adjustment for use in assessment of 30-day readmission as a hospital quality indicator is not straightforward. In this paper, we evaluate the adequacy of risk adjustment by comparing the one-year costs of those readmitted within 30 days to those not after excluding the costs of the readmission.

**Methods:**

In this two-step study, we first used comprehensive administrative and survey data on a nationally representative Medicare cohort of hospitalized patients to compare patients with a medical admission who experienced a 30-day readmission to patients without a readmission in terms of their overall Medicare payments during 12 months following the index discharge. We then examined the extent to which a series of enhanced risk adjustment models incorporating code-based comorbidities, self-reported health status and prior healthcare utilization, reduced the payment differences between the admitted and not readmitted groups.

**Results:**

Our analytic cohort consisted 4684 index medical hospitalization of which 842 met the 30-day readmission criteria. Those readmitted were more likely to be older, White, sicker and with higher healthcare utilization in the previous year. The unadjusted subsequent one-year Medicare spending among those readmitted ($56,856) was 60% higher than that among the non-readmitted ($35,465). Even with enhanced risk adjustment, and across a variety of sensitivity analyses, one-year Medicare spending remained substantially higher (46.6%, *p* < 0.01) among readmitted patients.

**Conclusions:**

Enhanced risk adjustment models combining health status indicators from administrative and survey data with previous healthcare utilization are unable to substantially reduce the cost differences between those medical admission patients readmitted within 30 days and those not. The unmeasured patient severity that these cost differences most likely reflect raises the question of the fairness of programs that place large penalties on hospitals with higher than expected readmission rates.

**Electronic supplementary material:**

The online version of this article (10.1186/s12913-019-3983-7) contains supplementary material, which is available to authorized users.

## Background

Studies over several decades have emphasized the inadequacy of administrative data-based risk adjustment models like that used in the US by the Centers for Medicare and Medicaid Services (CMS) in its 30-day hospital readmission profiling and penalty program, largely because administrative databases include only limited information on patient severity and disease burden [1–7]. Despite the criticism, there is broad consensus on the preventability of some readmissions [8] and evidence of reductions in readmissions from targeted interventions [9]. Thus, the CMS models and others like it continue to be in routine use to obtain publicly reported hospital measures of quality and performance-based penalties [10–13]. As an indication of the importance of these models, CMS imposed penalties estimated at $528 million on 78% of US hospitals in 2017 as part of the Hospital Readmissions Reduction Program [12] because of excess readmissions over those predicted by the risk adjustment model. In recent years, research papers have described models using additional variables from administrative databases (e.g., race/ethnicity and socio-economic status) as well as enhancing administrative data with self-reported and medical chart data, which capture previously unmeasured patient risk indicators such as health behavior, mental health status, functional health, socioeconomic vulnerabilities, and family and social support [2–4, 14–24]. However, the extent to which previously unmeasured disease burden and severity is captured in the enhanced models is unclear.

The value of 30-day readmissions as a performance measure depends upon the extent to which risk adjustment is able to “make comparable” at the time of the index hospital admission those that are readmitted within 30 days and those that are not. For, only if the two groups are comparable at the time of admission is it reasonable to penalize the hospital for the readmission. To evaluate the adequacy of risk adjustment when analyzing 30-day readmission rates, in this paper we take a novel approach that as far as we know has not been used before: specifically, we examine the longer term costs (one-year costs) of those readmitted within 30 days and those not after excluding the costs of the readmission. If risk adjusted longer-term costs are same in the readmitted and not readmitted groups, it suggests the groups are comparable with the exception of the readmission. This finding would provide strong support for the validity of 30-day readmissions as a performance measure. If longer-term costs differ between the two groups, there are two possible explanations: 1) a hospital error or deficiency in practice (e.g., inadequate discharge planning) has long-term cost implications; or, 2) there are still important unmeasured risk factors (e.g., unmeasured patient severity). To the extent costs associated with the first explanation tend to be of a short-term nature, longer-term differences in costs are an indication of inadequate risk adjustment. Also, whatever the cause of the difference in risk-adjusted longer-term costs between the admitted and not readmitted groups, if there is a significant difference, it becomes increasingly unreasonable to estimate the dollar savings from a reduction in readmissions as the costs of the readmission “prevented.” [25] It seems much more likely that due to the increased severity of the readmitted group, the “prevented” 30-day readmission was not really prevented but just shifted to a later time after 30 days.

### Risk prediction and risk adjustment models

Before turning to our specific study, we briefly place our work in a somewhat broader 30-day readmission risk modeling context. The literature on models that predict the likelihood of hospital readmissions can be distinguished based on purpose, which to a large extent dictates the variables available for modeling and thus, how well the model is likely to perform. **Risk prediction models** that attempt to identify patients at high risk for readmission during the course of their hospitalization can use variables whose values become available while the patient is in the hospital (e.g., days in the ICU); risk prediction models that prioritize patients for post-discharge interventions can use information available only at the time of discharge (e.g., length of stay). Much of the literature focusing on in-hospital and post-discharge interventions to reduce readmission risk has evaluated the extent to which laboratory data and vital signs, plus additional data from electronic health records, can improve the ability of the model to better identify high risk patients and target them for interventions. Though **risk adjustment models** predict risk for individual patients, that is not their goal. It is to control (or adjust) for differences in patient characteristics, essentially “leveling the playing field,” so that when the outcome of one group of patients (e.g., those treated at particular hospital or those receiving a particular intervention) is compared to another group, it is under the assumption that both groups are similar prior to event of interest (e.g., admission to the hospital or receipt of the intervention). When the models are used for provider profiling or incentive programs, which are usually undertaken by large administrative units (e.g., states, provinces or countries), the models are limited to data from administrative databases in which data elements are collected in standardized ways across a large number of provider. In addition, these models do not use data that may create perverse incentives, e.g., prior utilization or cost. However, when risk adjustment models are used to evaluate an intervention, the important variables included in the model are confounders, i.e., variables related both to receipt of the intervention and, independent of the intervention, to the outcome. If important confounders are not controlled for, it is impossible to know if an outcome following an intervention is due to the intervention or the uncontrolled for confounders. For example, prior utilization may increase the likelihood a patient receives a particular intervention and, because prior utilization may be associated with increased illness burden, it is likely related to the outcome whether or not the person receives the intervention.

In this paper, we initially consider a risk adjustment model with independent variables similar to the ones used by CMS to predict 30-day readmissions. These models, which include age, sex and comorbidities from CMS administrative databases, have c statistics (a standard measure of model performance when predicting a binary outcome variable) in the low 0.60 range (often considered below the “acceptable discrimination” threshold of 0.70) [26]. Models using prior utilization and data available at the time of hospital discharge to predict 30-day readmissions can have c statistics above 0.80 [3].

In what follows, we sequentially do the following: 1) Compare one-year subsequent healthcare utilization (measured as Medicare payments) between 30-day readmitted and non-readmitted medical admission patients, excluding payments for the 30-day readmission stay of the readmitted patients; 2) Examine the extent to which the large differences in healthcare utilization (our outcome) between the readmitted and non-readmitted group (essentially, the intervention, which is passive and sorts patients into 2 groups) could be “explained” by including additional variables (potential confounders) in the risk adjustment model; and 3) Finally, in the Discussion, pull together information from the analyses that provides support for the hypothesis that unmeasured disease burden (an unmeasured confounder) is the most likely factor accounting for the still large differences in costs that remain after controlling for a wide range of factors. In these analyses, we used longitudinal healthcare utilization data from CMS’ Medicare Current Beneficiary Survey (MCBS), which includes both administrative and survey data and therefore permits evaluation of a range of patient risk factors beyond those identified in administrative data [3, 15].

## Methods

### Data and analytic sample

We used 2000–2011 MCBS Cost and Use files. The MCBS is a weighted stratified, multistage, area probability sample of Medicare enrollees (community and facility dwellers) drawn from the Medicare enrollment file [27]. The sample cohort consists of three rotating panels, each followed for 3 years, with one panel replaced each year. Medicare claims data are supplemented with individual surveys of demographics, health status, health behavior, healthcare utilization and Medicare payments.

There were 38,059 enrollees aged 65 and older with claims data for 3 years, or until death during the study period. We excluded those enrolled in any Medicare Advantage plan during their three-year survey period (*n* = 391) (since claims data are unavailable for this group), who were residents of Puerto Rico (*n* = 36), and with missing key measures (*n* = 195). Further details on exclusions are in the Additional file 1.

In identifying index hospitalizations, we included all non-surgical hospitalizations, identified by Diagnostic Related Groups (DRG) designation of “medical” [28]. We look at 30-day readmissions occurring after all these non-surgical hospitalizations. We wanted to ensure at least 12 months of follow-up healthcare utilization after all index hospitalizations. Therefore, we selected the first hospitalization during the second year of the follow-up period of the 3-year MCBS cohort as index hospitalizations; those without a hospitalization in the second year were excluded from the analysis. Following previous work on evaluation of 30-day readmissions as a quality indicator, we excluded index hospitalizations that involved patient death within 30-days of index discharge, transfer to another acute care facility, discharge against medical advice or discharge to hospice [29].

### Study design

To examine if hospitalized patients with a 30-day readmission (“readmitted patients”) had different healthcare spending and utilization patterns compared to those without a 30-day readmission (“non-readmitted patients”), we used a retrospective study design to compare total healthcare spending following an index hospitalization (excluding costs associated with a readmission) between patients with and without a 30-day readmission.

### Outcomes

Our main outcome measure was one-year subsequent Medicare spending ($), defined as the total Medicare spending for all inpatient and outpatient care during one year after the index hospitalization admission date. For clarity in comparison, we excluded Medicare payments for the index inpatient stay as well as the payment of readmission stay for the subgroup with a 30-day readmission; all other hospitalizations during the 12-month follow-up period were included. We used Medicare payments reported in each claim record as the measure of healthcare spending. For comparability of spending over time, we applied the national Consumer Price Index to express all dollar values in terms of 2011 dollars [30]. To limit the influence of outliers, we top-coded individual annual spending at the 95th percentile (with larger spending values reset to the 95th percentile level, which is $200,000).

As secondary outcomes, we examined Medicare spending and utilization one year following discharge from the index admission by type of service: acute inpatient care spending; number of days in acute inpatient care; outpatient care spending; had an outpatient care visit within 30 days of index discharge. Utilization and payments associated with the readmission stay (for those readmitted) were not included in any measure.

### Independent variables

The main independent variable of interest was 30-day readmission status (Yes = 1, No = 0, indicated by readmission to any hospital for any admission condition within 30 days after the index discharge date. We included a range of other independent variables, clinical and non-clinical, identified as risk factors for 30-day readmission in prior work [3, 7, 15]. These included patients’ sex, age and race/ethnicity (non-Hispanic Whites, non-Hispanic Blacks, Hispanics, Others). As our study cohort includes all inpatient admissions, we used the Charlson Comorbidity Index conditions, coded as indicator variables [31]. Comorbidity condition status was based on (a) all secondary diagnosis codes in the index admission and (b) all diagnosis codes in the inpatient and outpatient records one-year prior to the index admission date.

Following prior studies using supplemental data, we also identified a range of self-reported patient health behavior and other risk factors [3, 7, 15]: ever smoking; overweight and obesity (body mass index > 25); living type (community-alone, community-two people, community-more than two and facility); education (< 12 years of education, high school, college); income categorized based on quartiles as lowest, second lowest and top two quartiles; marital status; and Medicaid coverage in addition to Medicare (dual coverage).

We included three measures of healthcare utilization during the one-year period prior to the index hospitalization: number of hospitalizations; total days of inpatient stay; and overall Medicare spending. To control for secular trends in health care costs other than due to inflation (which we adjusted for using the Consumer Price Index), we adjusted for calendar year of the index hospitalization. To adjust for systematic regional variation in healthcare utilization, we used enrollee’s residence location which, based on the Dartmouth hospital referral region-level measure of Medicare spending per person, was assigned to quintile of per-person spending [32]. We also adjusted for the following admission conditions: heart failure, pneumonia, pulmonary disease, digest disorder, gastrointestinal bleeding, septicemia, psychoses, intracranial hemorrhage/cerebral infarction, kidney & urinary tract infections, circulatory disorders, and other conditions.

### Statistical analysis

We performed bivariate comparisons of the aforementioned covariates between readmitted and non-readmitted patients using regression models – binary logit, multinomial logit and ordinary least squares, depending on the covariate measure – in order to most easily adjust for survey weights. For our core analysis of the comparison of the main outcome (subsequent one-year Medicare spending) between readmitted vs. non-readmitted patients, we estimated ordinary least squares (OLS) models of subsequent Medicare spending including readmission status and covariates as independent variables, again using survey weights. To adjust for skewness in the outcome measure, and following prior studies, we also estimated a generalized linear model (GLM) with a gamma distribution and log link; as estimates from both models were similar, to facilitate direct interpretation of coefficients, we have reported the OLS estimates as our preferred results (GLM estimates are in the Additional file 1). We estimated four models with different combinations of covariates. Model 1 patient sex, age, race/ethnicity, smoking status, overweight status, comorbidities, dual coverage, Hospital Referral Region-level Medicare spending (quintiles), index admission condition, census region (*N* = 9) and index year (2001–2010). Model 2 also included patient education, income, living type, and marital status. Model 3 included only indicators of prior year patient care (i.e., hospitalizations days, number of hospitalizations, overall medical spending in the prior years). Model 4, the most comprehensive model, extended Model 2 by including prior year patient care measures. All model estimates were based on heteroskedasticity-consistent robust standard errors. All analyses were conducted using Stata 13 (StataCorp, College Station, Texas).

In addition to the GLM models, we performed other sensitivity analyses (all reported in the Additional file 1). First, because systematic differences in one or more of the model covariates between readmitted vs. non-readmitted patients may potentially influence final estimates, we repeated the analysis with a propensity score matched sample of readmitted and non-readmitted cases. To create the matched samples, we used the following approach suggested by Austin [33]: randomize all readmitted cases; set as caliper 0.5 of the standard deviation of the logit of the propensity score (this was the smallest caliper size that ensured at least two matched observations for each readmitted case); select in order of the randomized readmitted cases the first two non-readmitted cases within the caliper distance of the readmitted cases without replacement; run an ordinary least square regression model with these matched groups. Standard errors that included error in estimation of the propensity score were used. To evaluate the success of matching, we compared covariate balance between readmitted and matched non-readmitted cases. When there are survey weights, there are a number of unresolved issues when matching on propensity scores [33]. Thus, we ran propensity score analysis without adjusting for survey weights. As an indirect test of the sensitivity of estimates to survey weight adjustment, we compared our main OLS estimates with and without survey weights. Second, systematic differences in patient death between readmitted vs. non-readmitted patients may influence subsequent healthcare utilization. To test the sensitivity of results to this possibility, we estimated out main model (OLS) for the subgroup of patients who did not die during the 3-year survey period. Finally, it may be that overall readmitted patients have higher one-year spending than non-readmitted patients, but the difference is driven by the higher proportion of very expensive cases among the readmitted patients. To evaluate this possibility, we examined the difference in one-year costs of readmitted and non-readmitted patients who had spending that was below different dollar thresholds.

## Results

Our analytic cohort consisted of 4684 index hospitalizations of which 842 met the 30-day readmission criteria. Although similar in some characteristics (see Table 1), those readmitted were more likely than the non-readmitted to be older, White, sicker (Charlson comorbidity), covered by Medicaid (dual coverage), and with higher healthcare utilization in the previous year. The average subsequent one-year Medicare spending was $39,314 overall; among the readmitted, average spending was $56,856, over 60% higher than among the non-readmitted, $35,465.

**Table 1:** Sample Characteristics of Studied Population (*N* = 4684)

	*N*		%		
	Readmission	No Readmission	Readmission	No Readmission	*P* values
All	842	3842	18.0	82.0	
Female	483	2204	57.4	57.4	0.988
Age					0.064
65–74	297	1564	35.3	40.7	
75–84	405	1588	48.1	41.3	
85+	140	691	16.6	18.0	
Race/Ethnicity					0.003
Whites	682	3280	81.0	85.4	
Blacks	87	294	10.3	7.7	
Hispanics	34	134	4.0	3.5	
Charlson comorbidity categories					
Acute myocardial infarction (AMI)	100	491	11.9	12.8	0.551
Congestive heart failure (CHF)	302	1111	35.9	28.9	< 0.001
Peripheral vascular disease (PVD)	183	697	21.7	18.1	0.032
Cerebrovascular disease	268	1002	31.8	26.1	0.001
Dementia	62	242	7.4	6.3	0.220
Chronic Obstructive Pulmonary Disease (COPD)	341	1377	40.5	35.8	0.025
Rheumatoid Disease	42	232	5.0	6.0	0.272
Peptic Ulcer	44	176	5.2	4.6	0.463
Mild liver disorder	17	24	2.0	0.6	0.001
Diabetes	330	1295	39.2	33.7	0.002
Diabetes + Complications	92	399	10.9	10.4	0.662
Hemiplegia or Paraplegia	29	64	3.5	1.7	0.002
Renal disease	137	483	16.2	12.6	0.011
Cancer	192	721	22.8	18.8	0.006
Moderate/severe Liver disease	9	15	1.1	0.4	0.024
Metastic Cancer	60	140	7.1	3.6	< 0.001
Acquired immune deficiency syndrome (AIDS)	1	3	0.1	0.1	0.736
Ever-smoker	499	2220	59.3	57.8	0.458
Overweight/Obese	496	2360	59.0	61.4	0.204
Education					0.175
< 12 years of education	299	1252	35.5	32.6	
High school	428	2040	50.8	53.1	
College and above	115	550	13.6	14.3	
Income, Household					0.104
Poorest quartile	172	699	20.5	18.2	
Second quartile	249	1099	29.6	28.6	
Top two quartiles	421	2044	50.0	53.2	
Marital Status					0.301
Married	385	1849	45.8	48.1	
Widowed	358	1552	42.5	40.4	
Divorced/separated	74	316	8.8	8.2	
Never married	25	125	3.0	3.2	
Living Type					0.952
Community-Alone	281	1285	33.4	33.4	
Community-Two people	401	1847	47.6	48.1	
Community-More than two	131	530	15.6	13.8	
Facility	29	180	3.5	4.7	
Dual (Medicare-Medicaid) coverage	159	583	18.9	15.2	0.006
Death During Follow-up	278	740	33.0	19.3	< 0.001
Utilizations by hospital referral regions					0.009
First quintile	100	610	11.8	15.9	
Second quintile	137	625	16.3	16.3	
Third quintile	137	700	16.2	18.2	
Fourth quintile	255	1057	30.3	27.5	
Fifth quintile	213	851	25.4	22.1	
Previous year utilization					
Days of stay	4.6	2.4	4.6	2.4	< 0.001
Number of hospitalizations	0.7	0.4	0.7	0.4	< 0.001
Overall Medicare spending ($)	39,004.5	25,103.7	39,004.5	25,103.7	< 0.001
Medicare spending 1-year following index discharge	56,855.5	35,464.5	56,855.5	35,464.5	< 0.001

*Notes.* Index condition, region and year are not included in this table

Adjusted for risk factors in Model 1, subsequent one-year Medicare spending among the readmitted patients was $17,726 (50%) higher than that among the non-readmitted (Table 2). Age group 65–74, Black race, and comorbidities of renal disorders, diabetes, and peripheral vascular disease were associated with higher spending. Adjusting further for patients’ nonclinical factors led to no sizable change in this difference (Model 2, Table 2). An alternate model which only adjusted for indicators of inpatient care utilization in the previous year, led to an adjusted difference of $18,163 (51%) in spending between those with and without readmission (Model 3, Table 2). Our final model that adjusted for all aforementioned covariates indicated a $16,516 (47%) difference in spending (Model 4, Table 2 and Additional file 1: Table S2).

**Table 2:** Models of Medicare spending 1-year following index discharge (*N* = 4684)

	Model 1	Model 2	Model 3	Model 4
Readmission	17726***	17670***	18163***	16516***
Female	524	894		413
Age				
65–74	Reference	Reference		Reference
75–84	− 872	−520		799
85+	− 8009***	− 7460**		− 4510^
Race				
Whites	Reference	Reference		Reference
Blacks	10750***	10079***		8768**
Hispanics	6619^	5677		6288
Others	− 2463	− 3586		− 2035
Dual (Medicare-Medicaid) coverage	− 1403	− 1836		− 2188
Ever Smoker	2703^	2687^		2689^
Overweight+Obese	− 2252	− 2432		− 1194
Education				
< 12 years of education		Reference		Reference
High school		− 1433		− 1534
Bachelor and above		− 4562^		− 4630^
Living Type				
Community-Alone		Reference		Reference
Community-Two people		1143		1411
Community-More than two		5162		4767
Facility		1228		2200
Income				
Poorest quartile		Reference		Reference
Second quartile		− 2059		− 2083
Top two quartiles		149		332
Marital Status				
Married		Reference		Reference
Widowed		− 824		38
Divorced/separated		559		1697
Never married		− 5076		− 3735
Previous year utilization				
Hospitalizations days			− 441^	− 343
Number of hospitalizations			2113	712
Overall Medicare spending ($)			0.255***	0.198***
Constant	6054	7821	29190***	8961
R-squared	0.13	0.14	0.11	0.17

*Notes*. Other covariates included in models 1, 2, and 4 were Charlson comorbidity index conditions, HRR-level Medicare spending (quintiles), index admission condition, census region (*N* = 9) and index year (2001–2010). All spending are in dollars

^ *p* < 0.10, ** *p* < 0.01, *** *p* < 0.001

Analogous comparison of secondary outcomes (using Model 4) indicated that the readmitted, compared to the non-readmitted, spent $13,191 more on acute inpatient care, were in inpatient care 12.6 more days, spent $3325 more on outpatient care, and had 17% higher likelihood of having an outpatient care visit within 30 days of index discharge (Table 3).

**Table 3:** Models of Medicare spending 1-year following index admission by type of service (*N* = 4684)

	(1)	(2)	(3)	(4)
	Acute inpatient care spending ($)	Acute inpatient care, days	Outpatient care spending ($)	Had an outpatient care visit within 30 days of index discharge
Average observed value	24,633	7.10	13,784	0.95
Model-predicted value of the difference in Medicare utilization associated with patients with 30-day readmission	13,191^***^	12.59^***^	3325^***^	0.17^***^
N	4684	4684	4684	4684
r2	0.14	0.23	0.19	0.04

*** *p* < 0.001

Our results were robust to alternative model specification (Additional file 1: Table S3). Using a generalized linear model with gamma distribution (instead of OLS model for Model 4, Table 2), subsequent one-year Medicare spending was $17,281 higher than among the non-readmitted. Matching readmitted with the non-readmitted based on propensity scores led to better balancing of all characteristics, including the proportion of Blacks and Medicaid covered, and previous year inpatient care (Additional file 1: Table S1). Regression estimation of the propensity score matched cohort indicated that spending among the readmitted was $18,337 higher than among those not readmitted (Additional file 1: Table S3). Also, the aforementioned excess spending estimate of $16,516 in Table 2 (Model 4) was not sensitive to use of survey weights; re-estimation without weights resulted in $16,281 excess spending (Additional file 1: Table S3). Our results were also robust after limiting the sample only to those who were alive throughout our three-year observation window ($19,945 difference in cost, Additional file 1: Table S4). As shown in Table 4, though the ratio of readmitted to non-readmitted spending declines as the threshold (i.e., the dollar amount below which patients are included in the average) is reduced from $130,000 to $15,000 (approximately the median spending of non-readmitted patients), low-cost readmitted patients still have between 28 to 50% higher costs than low-cost non-readmitted patients.

**Table 4:** Average Cost and Percent of Cases Below the Threshold: Readmitted and Non-Readmitted Patients

	Non-Readmitted Cases (NRC)		Readmitted Cases (RC)		Ratio of Average
Threshold $	Average Cost	% Cases	Average Cost	% Cases	Cost NRC to RC
130,000	23,718	0.93	35,558	0.86	1.50
120,000	22,637	0.92	33,679	0.84	1.49
110,000	21,717	0.91	32,179	0.83	1.48
100,000	20,684	0.90	29,467	0.80	1.42
90,000	19,395	0.88	27,875	0.78	1.44
80,000	17,947	0.87	25,453	0.75	1.42
70,000	16,601	0.85	24,183	0.73	1.46
60,000	15,006	0.82	21,256	0.68	1.42
50,000	13,264	0.78	18,022	0.62	1.36
40,000	11,551	0.74	14,745	0.55	1.28
30,000	9264	0.68	12,376	0.49	1.34
20,000	7045	0.59	9164	0.39	1.30
15,000	5740	0.53	7368	0.32	1.28

## Discussion

Across a number of different models and sensitivity analyses, we consistently found that medical admission patients readmitted within 30 days have approximately 50% higher one-year costs than those not readmitted. In particular, enhanced risk adjustment had no major effect on the cost differences between the two groups. This gives rise to the question “what accounts for the difference?” In some cases a serious medical error in the initial hospitalization of those readmitted within 30 days may have led to substantially higher utilization and costs over the subsequent year. However, there is nothing in the literature of which we are aware that suggests most 30-day readmissions are due to serious medical errors with long-term cost implications at the index hospitalization. It is also possible that the higher death rate among those readmitted combined with high end-of-life utilization patterns accounts for the difference in payments between the readmitted and not readmitted groups. However, as noted, when we reran the models including only those who survived for the full 3-year period, we still found substantial differences between those readmitted and those not ($19,945 difference in costs, Additional file 1: Table S4).

Another possibility is that there may be systematic differences in provider practices between the two cohorts in terms of the risk of admission and readmission; for instance, some geographic areas may have lower thresholds, in terms of patient severity, in admitting and readmitting patients [34]. Note, however, that we have controlled for patients’ healthcare spending in the previous year. To illustrate the implications of this, consider two patients with equally high health care spending in the year prior to their index admission (which the model allows us to do, since it includes prior health care spending as an independent variable). Assume that in terms of unmeasured patient severity the two patients are similar to the average patients in the sample and that their high spending is due to the fact that the patients live in areas with an equally high propensity to consume health care resources (i.e., low threshold to provide services). One of the patients has a 30-day readmission and the other patient does not. Assume one-year costs of the readmitted patient (excluding the readmission) are 50% higher than the non-readmitted patient. It is certainly possible that the providers for the readmitted patient have increased even further their propensity to provide services from the pre-period whereas those for the non-readmitted patient have not. However, the more likely hypothesis is that the readmitted patient became sicker than the non-readmitted patient and this increased illness burden is the reason for the readmission and the higher one-year costs. Several facts support our hypothesis: 1) In addition to controlling for one-year prior health care spending, we have also controlled for the average annual Medicare spending per enrollee in the patient’s hospital referral region; 2) Readmitted patients not only had 61.0% higher inpatient care spending but 25.6% higher adjusted outpatient care spending in the subsequent one year period following the index hospitalization; and they were 17% more likely to have an outpatient visit within 30 days of the index hospitalization discharge; 3) Observed risk factors were more prevalent among the readmission cohort; in particular, readmitted patients had higher prevalence of heart failure, cerebrovascular disease, chronic obstructive pulmonary disease, dementia, renal disease and cancer; and finally, 4) The death rate among readmitted patients was substantially higher than among non-readmitted patients (33% vs. 19%).

A possible bias in our analysis resulting from exclusion of the costs of the readmission is that those readmitted had less days (i.e., the time they were in the hospital) to experience outpatient costs than those not readmitted. Thus, our approach may underestimate the cost differences between the two groups. To examine this, we reran the analysis after eliminating costs in the first 30 days after discharge for both groups. The change was not in the expected direction, i.e., a larger difference between the 2 groups. In the original analysis, the cost difference was $16,516; in the suggested analysis that looks at cost differences starting from day 30 after the index admission, the cost difference was $13,850. The reason for this is that when we eliminate the first 30 days after discharge from the index admission, we eliminate a period of time during which, in addition to the readmission, the readmitted group has much higher outpatient healthcare utilization. For example, the readmitted group had 12.4 more physician visits in the 30-days post discharge than the non-readmitted.

Studies have indicated a reduction in readmission rates nationwide following CMS’ introduction of annual reporting of hospital performance in readmissions (Hospital Compare program) and CMS’ penalty program for excess readmissions (Hospital Readmissions Reduction Program) [35–37]. A recent study, however, suggests that the impact on readmissions in prior studies is at a minimum only half that previously estimated and at a maximum statistically similar to the declines in two control samples [38, 39]. As our study was based predominantly on data prior to these programs, it would be useful to use examine the robustness of our findings using more recent data, something feasible since the CMS methodology for estimation of risk-adjusted readmissions performance has largely remained unchanged.

We recognize several limitations of this study. Because our study population was limited to a small sample of Medicare participants with few index hospitalizations from the same hospitals, we were unable to examine hospital-level differences in readmissions. Also, our choice of additional variables measuring readmission risk were limited to those available in MCBS data; however, we were able to identify measures covering most of the domains covered in previous studies [3]. On the positive side, because the MCBS sample is a stratified national sample of Medicare enrollees, we expect that the findings are representative of patients across all hospitals.

## Conclusions

In summary, our study suggests that on average readmitted patients are “sicker” than non-readmitted patients and that current models of risk adjustment for 30-day readmission, even when supplemented with self-reported measures of patient health behavior, functional health status, family and social support, prior utilization and socioeconomic disadvantage, are unable to adjust for these differences. Therefore, use of such models for profiling hospital performance on 30-day readmission may systematically underestimate performance of hospitals with high rates of observed readmissions. Finally, our findings do call in to question studies that have estimated the benefits of a reduction in 30-day readmissions as “true” savings to the health care system. Given the likely increased morbidity and disease severity of the readmitted group, it seems probable that many of the “prevented” 30-readmissions will be readmitted after the 30-day period.

## Additional file

Additional file 1: **Table S1.** Comparison of sample characteristics before vs. after propensity score matching. **Table S2.** Models of Medicare spending 1-year following index discharge (*N* = 4684). **Table S3.** Sensitivity of estimates to model functional form: Overall Medicare spending 1-year following index discharge (*N* = 4684). **Table S4.** Sensitivity of estimates to exclusions: Overall Medicare spending 1-year following index discharge. Exclusions B1. Keeping Eligible Admissions in the Second Year. Exclusions B2. Other Exclusions. (DOCX 36 kb)

## Abbreviations

CMS: Centers of Medicare & Medicaid Services
DRG: Diagnostic Related Groups
GLM: Generalized Linear Model
MCBS: Medicare Current Beneficiary Survey
OLS: Ordinary Least Squares
