# Supplementary material for: One-year costs of medical admissions with and without a 30-day readmission and enhanced risk adjustment
Source: BMC Health Serv Res. 2019 Mar 12;19:155. doi: 10.1186/s12913-019-3983-7 (PMC6416984; doi:10.1186/s12913-019-3983-7)
Supplement: Supplementary file 1 — Table S1. Comparison of sample characteristics before vs. after propensity score matching. Table S2. Models of Medicare spending 1-year following index discharge (N = 4684). Table S3. Sensitivity of estimates to model functional form: Overall Medicare spending 1-year following index discharge (N = 4684). Table S4. Sensitivity of estimates to exclusions: Overall Medicare spending 1-year following index discharge. Exclusions B1. Keeping Eligible Admissions in the Second Year. Exclusions B2. Other Exclusions. (DOCX 36 kb) [file 12913_2019_3983_MOESM1_ESM.docx]

Additional file 1

[Table A1. Comparison of sample characteristics before vs. after propensity score matching 2](#_Toc494385018)

[Table A2. Models of Medicare spending 1-year following index discharge (N=4684) 4](#_Toc494385019)

[Table A3. Sensitivity of estimates to model functional form: Overall Medicare spending 1-year following index discharge (N=4684) 7](#_Toc494385020)

[Table A4. Sensitivity of estimates to exclusions: Overall Medicare spending 1-year following index discharge 8](#_Toc494385021)

[Exclusions B1. Keeping Eligible Admissions in the Second Year 9](#_Toc494385022)

[Exclusions B2. Other Exclusions 9](#_Toc494385023)

| Table S1. Comparison of sample characteristics before vs. after propensity score matching | | | | |  |
| --- | --- | --- | --- | --- | --- |
|  | Unmatched | Mean | | t-test | |
| Variable | Matched | Treated | Control | t | p>t |
| Female | U | 57.9% | 58.0% | -0.01 | 0.992 |
|  | M | 57.9% | 56.6% | 0.54 | 0.587 |
| Age (%) |  |  |  |  |  |
| 75-84 | U | 49.8% | 43.9% | 3.15 | 0.002 |
|  | M | 49.8% | 50.8% | -0.39 | 0.696 |
| 85+ | U | 20.4% | 22.0% | -0.98 | 0.328 |
|  | M | 20.4% | 20.0% | 0.24 | 0.808 |
| Race (%) |  |  |  |  |  |
| Blacks | U | 10.3% | 7.8% | 2.32 | 0.020 |
|  | M | 10.3% | 10.6% | -0.24 | 0.811 |
| Hispanics | U | 4.1% | 3.7% | 0.47 | 0.640 |
|  | M | 4.1% | 3.8% | 0.25 | 0.802 |
| Others | U | 4.1% | 3.4% | 1.01 | 0.314 |
|  | M | 4.1% | 4.1% | 0.00 | 1.000 |
| Dual (Medicare-Medicaid) coverage | U | 20.0% | 16.9% | 2.10 | 0.036 |
|  | M | 20.0% | 19.7% | 0.12 | 0.903 |
| Ever Smoker | U | 57.6% | 56.0% | 0.83 | 0.406 |
|  | M | 57.6% | 57.1% | 0.20 | 0.843 |
| Overweight+Obese | U | 57.2% | 59.6% | -1.25 | 0.210 |
|  | M | 57.2% | 57.0% | 0.10 | 0.921 |
| Charlson comorbidity categories (%) |  |  |  |  |  |
| Acute myocardial infarction (AMI) | U | 11.8% | 12.8% | -0.79 | 0.429 |
|  | M | 11.8% | 11.7% | 0.08 | 0.940 |
| Congestive heart failure (CHF) | U | 36.6% | 30.1% | 3.65 | <0.001 |
|  | M | 36.6% | 35.8% | 0.30 | 0.760 |
| Peripheral vascular disease (PVD) | U | 21.5% | 18.4% | 2.09 | 0.036 |
|  | M | 21.5% | 21.3% | 0.12 | 0.905 |
| Cerebrovascular disease | U | 31.8% | 26.7% | 3.01 | 0.003 |
|  | M | 31.8% | 31.3% | 0.21 | 0.833 |
| Dementia | U | 8.2% | 7.3% | 0.98 | 0.327 |
|  | M | 8.2% | 8.1% | 0.09 | 0.929 |
| Chronic Obstructive Pulmonary Disease (COPD) | U | 39.5% | 35.5% | 2.19 | 0.028 |
|  | M | 39.5% | 37.4% | 0.90 | 0.366 |
| Rheumatoid Disease | U | 4.7% | 6.0% | -1.47 | 0.142 |
|  | M | 4.7% | 5.1% | -0.45 | 0.651 |
| Peptic Ulcer | U | 4.3% | 4.7% | -0.48 | 0.632 |
|  | M | 4.3% | 5.3% | -0.92 | 0.360 |
| Mild liver disorder | U | 1.8% | 0.7% | 3.25 | 0.001 |
|  | M | 1.8% | 1.1% | 1.23 | 0.218 |
| Diabetes | U | 39.1% | 33.0% | 3.36 | 0.001 |
|  | M | 39.1% | 40.3% | -0.50 | 0.618 |
| Diabetes + Complications | U | 10.6% | 9.7% | 0.86 | 0.390 |
|  | M | 10.6% | 11.9% | -0.85 | 0.396 |
| Hemiplegia or Paraplegia | U | 3.5% | 1.8% | 2.99 | 0.003 |
|  | M | 3.5% | 3.6% | -0.13 | 0.895 |
| Renal disease | U | 15.9% | 12.3% | 2.77 | 0.006 |
|  | M | 15.9% | 17.6% | -0.92 | 0.360 |
| Cancer | U | 21.5% | 18.5% | 2.00 | 0.045 |
|  | M | 21.5% | 22.9% | -0.71 | 0.481 |
| Moderate/severe Liver disease | U | 1.0% | 0.4% | 2.27 | 0.023 |
|  | M | 1.0% | 0.8% | 0.26 | 0.796 |
| Metastic Cancer | U | 6.0% | 3.6% | 3.18 | 0.001 |
|  | M | 6.0% | 6.7% | -0.60 | 0.547 |
| Acquired immune deficiency syndrome (AIDS) | U | 0.1% | 0.1% | 0.37 | 0.711 |
|  | M | 0.1% | 0.2% | -0.58 | 0.564 |
| Education (%) |  |  |  |  |  |
| High school | U | 49.9% | 51.7% | -0.93 | 0.351 |
|  | M | 49.9% | 52.3% | -0.98 | 0.328 |
| College and above | U | 13.3% | 13.5% | -0.19 | 0.850 |
|  | M | 13.3% | 13.0% | 0.14 | 0.885 |
| Living Type (%) |  |  |  |  |  |
| Community-Two people | U | 45.8% | 46.1% | -0.20 | 0.838 |
|  | M | 45.8% | 45.8% | 0.00 | 1.000 |
| Community-More than two | U | 15.1% | 13.1% | 1.49 | 0.137 |
|  | M | 15.1% | 13.1% | 1.12 | 0.261 |
| Facility | U | 5.5% | 6.8% | -1.42 | 0.154 |
|  | M | 5.5% | 5.5% | 0.00 | 1.000 |
| Income, Household (%) |  |  |  |  |  |
| Second quartile | U | 30.6% | 29.8% | 0.46 | 0.643 |
|  | M | 30.6% | 29.3% | 0.59 | 0.557 |
| Top two quartiles | U | 47.7% | 50.2% | -1.33 | 0.183 |
|  | M | 47.7% | 49.7% | -0.83 | 0.406 |
| Marital Status (%) |  |  |  |  |  |
| Widowed | U | 44.3% | 43.1% | 0.65 | 0.518 |
|  | M | 44.3% | 44.0% | 0.15 | 0.883 |
| Divorced/separated | U | 8.1% | 7.8% | 0.36 | 0.720 |
|  | M | 8.1% | 7.9% | 0.18 | 0.857 |
| Never married | U | 3.0% | 3.5% | -0.69 | 0.491 |
|  | M | 3.0% | 3.2% | -0.28 | 0.778 |
| Previous year utilization |  |  |  |  |  |
| Days of stay | U | 4.07 | 2.38 | 5.68 | <0.001 |
|  | M | 4.07 | 4.09 | -0.05 | 0.963 |
| Number of hospitalizations | U | 0.68 | 0.44 | 5.97 | <0.001 |
|  | M | 0.68 | 0.67 | 0.18 | 0.854 |
| Overall Medicare spending ($) | U | 35330 | 23605 | 5.27 | <0.001 |
|  | M | 35330 | 37383 | -0.53 | 0.594 |
| *Notes.* 1) the results above didn't adjust for survey weight. 2) Caliper=0.5 SDs of the logit of the propensity score 3) Other covariates were HRR-level Medicare spending (quintiles), index admission condition, census region (N=9) and index year (2001-2010) | | | | | |

| Table S2. Models of Medicare spending 1-year following index discharge (N=4684) | | | | | | | | |
| --- | --- | --- | --- | --- | --- | --- | --- | --- |
|  | Model 1 |  | Model 2 |  | Model 3 |  | Model 4 |  |
| Readmission | 17726^***^ | [13639,21812] | 17670^***^ | [13606,21734] | 18163^***^ | [13843,22484] | 16516^***^ | [12294,20739] |
| Female | 524 | [-2612,3660] | 894 | [-2172,3960] |  |  | 413 | [-2418,3244] |
| Age |  |  |  |  |  |  |  |  |
| 65-74 | 0 | [0,0] | 0 | [0,0] |  |  | 0 | [0,0] |
| 75-84 | -872 | [-4343,2600] | -520 | [-4101,3061] |  |  | 799 | [-2485,4083] |
| 85+ | -8009^***^ | [-12279,-3739] | -7460^**^ | [-12290,-2630] |  |  | -4510^^^ | [-9106,86] |
| Race |  |  |  |  |  |  |  |  |
| Whites | 0 | [0,0] | 0 | [0,0] |  |  | 0 | [0,0] |
| Blacks | 10750^***^ | [4790,16709] | 10079^***^ | [4490,15667] |  |  | 8768^**^ | [3206,14329] |
| Hispanics | 6619^^^ | [-815,14053] | 5677 | [-1956,13310] |  |  | 6288 | [-1470,14046] |
| Others | -2463 | [-11941,7015] | -3586 | [-13114,5943] |  |  | -2035 | [-11423,7353] |
| Dual (Medicare-Medicaid) coverage | -1403 | [-5857,3051] | -1836 | [-6692,3021] |  |  | -2188 | [-6727,2350] |
| Ever Smoker | 2703^^^ | [-184,5591] | 2687^^^ | [-257,5631] |  |  | 2689^^^ | [-87,5465] |
| Overweight+Obese | -2252 | [-6219,1715] | -2432 | [-6458,1595] |  |  | -1194 | [-4920,2532] |
| Charlson comorbidity categories |  |  |  |  |  |  |  |  |
| Acute myocardial infarction (AMI) | 1020 | [-3857,5898] | 815 | [-4075,5705] |  |  | -2561 | [-7057,1934] |
| Congestive heart failure (CHF) | 6564^**^ | [2604,10524] | 6487^**^ | [2531,10444] |  |  | 3985^*^ | [129,7841] |
| Peripheral vascular disease (PVD) | 10981^***^ | [6858,15105] | 11107^***^ | [6942,15271] |  |  | 8887^***^ | [4584,13189] |
| Cerebrovascular disease | -1747 | [-5326,1832] | -1778 | [-5329,1773] |  |  | -3279^^^ | [-6757,199] |
| Dementia | 1842 | [-5376,9060] | 1522 | [-5898,8942] |  |  | 389 | [-6362,7139] |
| Chronic Obstructive Pulmonary Disease (COPD) | 6133^***^ | [3166,9100] | 5911^***^ | [2919,8903] |  |  | 4345^**^ | [1591,7099] |
| Rheumatoid Disease | 10696^**^ | [3046,18345] | 10741^**^ | [2942,18540] |  |  | 11047^**^ | [3339,18755] |
| Peptic Ulcer | 3344 | [-4302,10991] | 3395 | [-4223,11012] |  |  | 1111 | [-6228,8449] |
| Mild liver disorder | 9310 | [-14752,33372] | 8730 | [-15323,32782] |  |  | 7910 | [-17071,32891] |
| Diabetes | 5112^*^ | [1145,9079] | 4951^*^ | [986,8916] |  |  | 4687^*^ | [1057,8318] |
| Diabetes + Complications | 15768^***^ | [8178,23359] | 15590^***^ | [7997,23182] |  |  | 12596^**^ | [5160,20032] |
| Hemiplegia or Paraplegia | -4111 | [-15855,7634] | -4557 | [-16136,7022] |  |  | -6760 | [-17880,4360] |
| Renal disease | 17176^***^ | [11998,22355] | 17254^***^ | [12067,22441] |  |  | 11063^***^ | [5883,16243] |
| Cancer | 9324^***^ | [4839,13810] | 9451^***^ | [4992,13909] |  |  | 7755^***^ | [3395,12116] |
| Moderate/severe Liver disease | 1441 | [-28661,31542] | 2124 | [-28186,32434] |  |  | -761 | [-29645,28122] |
| Metastic Cancer | 11000^*^ | [923,21077] | 11079^*^ | [908,21250] |  |  | 4408 | [-5188,14004] |
| Acquired immune deficiency syndrome (AIDS) | 1435 | [-72412,75282] | 1860 | [-72023,75743] |  |  | 3986 | [-69226,77197] |
| Utilizations by hospital referral regions |  |  |  |  |  |  |  |  |
| First quintile | 0 | [0,0] | 0 | [0,0] |  |  | 0 | [0,0] |
| Second quintile | 5684^*^ | [448,10919] | 5713^*^ | [505,10920] |  |  | 5256^*^ | [440,10073] |
| Third quintile | 6267^*^ | [1497,11037] | 6278^*^ | [1506,11051] |  |  | 5749^*^ | [1228,10271] |
| Fourth quintile | 5892^*^ | [679,11104] | 5803^*^ | [527,11079] |  |  | 4808^^^ | [-300,9916] |
| Fifth quintile | 14510^***^ | [8363,20657] | 14379^***^ | [8263,20496] |  |  | 12957^***^ | [7129,18786] |
| Index condition |  |  |  |  |  |  |  |  |
| Heart Failure | 0 | [0,0] | 0 | [0,0] |  |  | 0 | [0,0] |
| Pneumonia | -4578 | [-16167,7011] | -4763 | [-16408,6882] |  |  | -3108 | [-13998,7781] |
| Pulmonary Disease | 1539 | [-11699,14777] | 1059 | [-12371,14489] |  |  | 4036 | [-8160,16232] |
| Digest Disorder | 9297 | [-5567,24162] | 9255 | [-5518,24029] |  |  | 10759 | [-4012,25529] |
| G.I. Hemorrhage | -5629 | [-18097,6839] | -6372 | [-18938,6194] |  |  | -5575 | [-17512,6361] |
| Septicemia | -6943 | [-22623,8738] | -7079 | [-22690,8532] |  |  | -7322 | [-21579,6934] |
| Psychoses | 2111 | [-21407,25630] | 1581 | [-22379,25541] |  |  | 3795 | [-18819,26408] |
| Intracranial Hemorrhage/Cerebral Infarction | -3497 | [-18384,11390] | -3717 | [-18567,11133] |  |  | -370 | [-14150,13410] |
| Kidney & Urinary Tract Infections | -4862 | [-17566,7842] | -5552 | [-18521,7416] |  |  | -2961 | [-15700,9778] |
| Circulatory Disorders | 5488 | [-13602,24578] | 4544 | [-14903,23991] |  |  | 7501 | [-12198,27199] |
| Others | -4527 | [-14039,4985] | -4589 | [-14089,4912] |  |  | -3186 | [-12092,5721] |
| Region |  |  |  |  |  |  |  |  |
| New England | 0 | [0,0] | 0 | [0,0] |  |  | 0 | [0,0] |
| Middle Atlantic | 7205 | [-2002,16411] | 6949 | [-2362,16260] |  |  | 5152 | [-3959,14263] |
| East North Central | 800 | [-8280,9880] | 368 | [-8740,9476] |  |  | -1743 | [-10607,7122] |
| West North Central | 5152 | [-4520,14825] | 4709 | [-5033,14452] |  |  | 2301 | [-7513,12115] |
| South Atlantic | 3965 | [-4834,12765] | 3207 | [-5645,12059] |  |  | 2015 | [-6562,10592] |
| East South Central | 4715 | [-5495,14926] | 4186 | [-6024,14395] |  |  | 2479 | [-7461,12419] |
| West South Central | 12049^*^ | [2026,22072] | 11543^*^ | [1597,21490] |  |  | 9055^^^ | [-830,18940] |
| Mountain | 12531^*^ | [1035,24027] | 12054^*^ | [535,23573] |  |  | 10027^^^ | [-1171,21225] |
| Pacific | 9698^^^ | [-1203,20600] | 9349^^^ | [-1554,20253] |  |  | 5015 | [-5559,15590] |
| Index year |  |  |  |  |  |  |  |  |
| 2001 | 0 | [0,0] | 0 | [0,0] |  |  | 0 | [0,0] |
| 2002 | 2394 | [-2352,7141] | 2277 | [-2444,6998] |  |  | 1343 | [-3241,5926] |
| 2003 | 6753^*^ | [769,12737] | 6632^*^ | [605,12660] |  |  | 5645^^^ | [-461,11750] |
| 2004 | 6395^*^ | [182,12608] | 6290^*^ | [61,12519] |  |  | 3942 | [-2242,10125] |
| 2005 | 8674^**^ | [3469,13878] | 8673^**^ | [3506,13839] |  |  | 6499^*^ | [1379,11620] |
| 2006 | 4210 | [-2163,10582] | 4191 | [-2087,10470] |  |  | 1388 | [-4574,7350] |
| 2007 | 7318^*^ | [1528,13108] | 7423^*^ | [1557,13290] |  |  | 5432^^^ | [-561,11424] |
| 2008 | 9871^**^ | [3625,16117] | 9623^**^ | [3280,15965] |  |  | 7384^*^ | [1285,13484] |
| 2009 | 10593^**^ | [3929,17258] | 10561^**^ | [3952,17170] |  |  | 8289^*^ | [1827,14751] |
| 2010 | 14243^***^ | [6951,21536] | 14171^***^ | [6852,21490] |  |  | 10286^**^ | [3246,17327] |
| Education |  |  |  |  |  |  |  |  |
| <12 years of education |  |  | 0 | [0,0] |  |  | 0 | [0,0] |
| High school |  |  | -1433 | [-5218,2352] |  |  | -1534 | [-5111,2044] |
| Bachelor and above |  |  | -4562^^^ | [-9810,687] |  |  | -4630^^^ | [-9607,346] |
| Living Type |  |  |  |  |  |  |  |  |
| Community-Alone |  |  | 0 | [0,0] |  |  | 0 | [0,0] |
| Community-Two people |  |  | 1143 | [-3611,5897] |  |  | 1411 | [-3173,5994] |
| Community-More than two |  |  | 5162 | [-1134,11458] |  |  | 4767 | [-1597,11132] |
| Facility |  |  | 1228 | [-6375,8832] |  |  | 2200 | [-4993,9393] |
| Income |  |  |  |  |  |  |  |  |
| Poorest quartile |  |  | 0 | [0,0] |  |  | 0 | [0,0] |
| Second quartile |  |  | -2059 | [-7217,3099] |  |  | -2083 | [-7078,2911] |
| Top two quartiles |  |  | 149 | [-5141,5438] |  |  | 332 | [-4706,5371] |
| Marital Status |  |  |  |  |  |  |  |  |
| Married |  |  | 0 | [0,0] |  |  | 0 | [0,0] |
| Widowed |  |  | -824 | [-5687,4040] |  |  | 38 | [-4664,4741] |
| Divorced/separated |  |  | 559 | [-6703,7820] |  |  | 1697 | [-5245,8638] |
| Never married |  |  | -5076 | [-14552,4399] |  |  | -3735 | [-13333,5864] |
| Previous year utilization |  |  |  |  |  |  |  |  |
| Days of stay |  |  |  |  | -441^^^ | [-967,85] | -343 | [-863,177] |
| Number of hospitalizations |  |  |  |  | 2113 | [-1145,5370] | 712 | [-2375,3798] |
| Overall Medicare spending ($) |  |  |  |  | 0^***^ | [0,0] | 0^***^ | [0,0] |
| Constant | 6054 | [-9961,22069] | 7821 | [-9438,25081] | 29190^***^ | [27477,30902] | 8961 | [-7427,25350] |
| R-squared | 0.13 |  | 0.14 |  | 0.11 |  | 0.17 |  |
| 95% confidence intervals in brackets  ^^^ *p* < 0.10, ^*^ *p* < 0.05, ^**^ *p* < 0.01, ^***^ *p* < 0.001 | | | | | | | | |

|  |  |  |  |
| --- | --- | --- | --- |
| Table S3. Sensitivity of estimates to model functional form: Overall Medicare spending 1-year following index discharge (N=4684) | | | |
|  | GLM-Gamma | Propensity Score Matching Without Adjusting for Survey Weights | OLS without adjusting for survey weights |
| Model-predicted value of the difference in Medicare utilization associated with patients with 30-day readmission | 17,281^***^ | 18,337^***^ | 16,281^***^ |
| *Notes.*The OLS model without adjusting for survey weights is identical to Table 2 Model 4 with the only difference that survey weights are not taken into account here. | | | |
| ^***^ *p* < 0.001 |  |  |  |

|  |  |  |  |
| --- | --- | --- | --- |
| Table S4. Sensitivity of estimates to exclusions: Overall Medicare spending 1-year following index discharge | | |  |
|  | Sample in the main analysis | Sample with only those who were alive throughout our three-year observation window |  |
| Model-predicted value of the difference in Medicare utilization associated with patients with 30-day readmission | 16,516*** | 19,945*** |  |
| N | 4684 | 3666 |  |
| ^***^ *p* < 0.001 |  |  |  |

|  |  |
| --- | --- |
| Exclusions B1. Keeping Eligible Admissions in the Second Year | |
| Out of the 38,059 cases, 14,729 had an inpatient admission and, of these, 12,167 had an admission in the second calendar year of their participation in the MCBS. Starting with 12,167 admissions, | |
| Exclude |  |
|  | - age group 0-64 (2,298 excluded) |
|  | - in-hospital death (492 excluded) |
|  | - transferred to another acute care facility (312 excluded) |
|  | - discharged against medical advice (20 excluded) |
|  | - discharged to hospice (176 excluded) |
| Ending with 8,869 admissions for 5,427 patients. | |
| Note that for transfers (defined as (1) within one-day transfer, (2) both stays had the same cohort event, and (3) both indicated transfer status), ideally we should treat them as one admission. Here, to simplify, we dropped the first record and use the second record from the transferred hospital. In this dataset, there were at most 35 such cases. | |
|  |  |
|  |  |
| Exclusions B2. Other Exclusions | |
| Starting with 5,427 patients who had eligible inpatient admission in the second year, | |
| Exclude |  |
|  | - patients who were ever/always enrolled in HMO during our three-year study period (391 excluded) |
|  | - patients who died within 30 days of index discharge (121 excluded) |
|  | - patient's residency is Puerto Rico (36 excluded) |
|  | - missing data in smoking (65 excluded) |
|  | - missing data in marital status (2 excluded) |
|  | - missing data in education (114 excluded) |
|  | - missing data in living type (11 excluded) |
|  | - missing data in Medicare spending 1-year following index discharge (3 excluded) |
| Ending with 4,684 patients. | |
